# Supplementary material for: Investigation of dermal collagen nanostructures in Ehlers-Danlos Syndrome (EDS) patients
Source: PLoS One. 2024 Aug 22;19(8):e0307442. doi: 10.1371/journal.pone.0307442 (PMC11341037; doi:10.1371/journal.pone.0307442)
Supplement: S1 File — (DOCX) [file pone.0307442.s001.docx]

**Analyzing regions marked by (*, **, ***) on hEDS-Scleroderma skin sample**

AFM images (5x5 µm) were acquired from three normal and three disrupted areas (guided by PS image) of the histological section of the skin biopsy. Subsequently, a total of 300 force curves were recorded for each of the normal and disrupted areas. A unimodal Yong’s moduli distribution was observed for both normal and disrupted collagen in all the three regions similar to hEDS-Scleroderma curves presented in Figure 3A. Suggesting that in each of these regions, a single population of collagen fibrils is present. These mechanical data must be interpreted carefully, as we only had one case with both EDS and scleroderma. Further association of the mechanical findings and H&E staining requires more skin tissue from similar patients


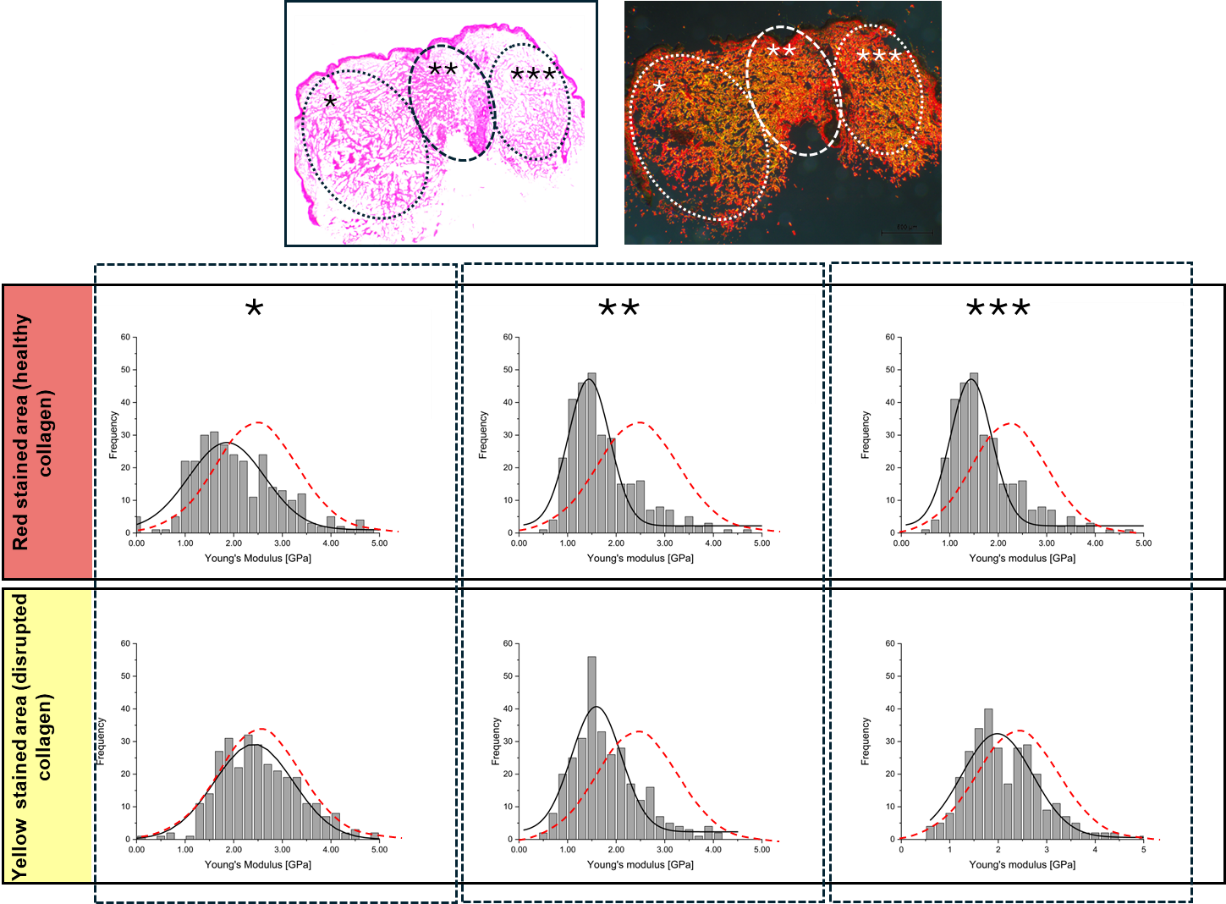


**Figure S1** Shows the distributions of Young's moduli of collagen fibrils recorded in the *, **, and ** regions of the hEDS-Scleroderma case. A unimodal distribution of Young's moduli was observed for both normal and disrupted collagen across all three regions. The red dotted line represents the distribution of healthy collagen in the control group
