# Supplementary figures and images for: Investigation of dermal collagen nanostructures in Ehlers-Danlos Syndrome (EDS) patients
Source: PLoS One. 2024 Aug 22;19(8):e0307442. doi: 10.1371/journal.pone.0307442 (PMC11341037; doi:10.1371/journal.pone.0307442)

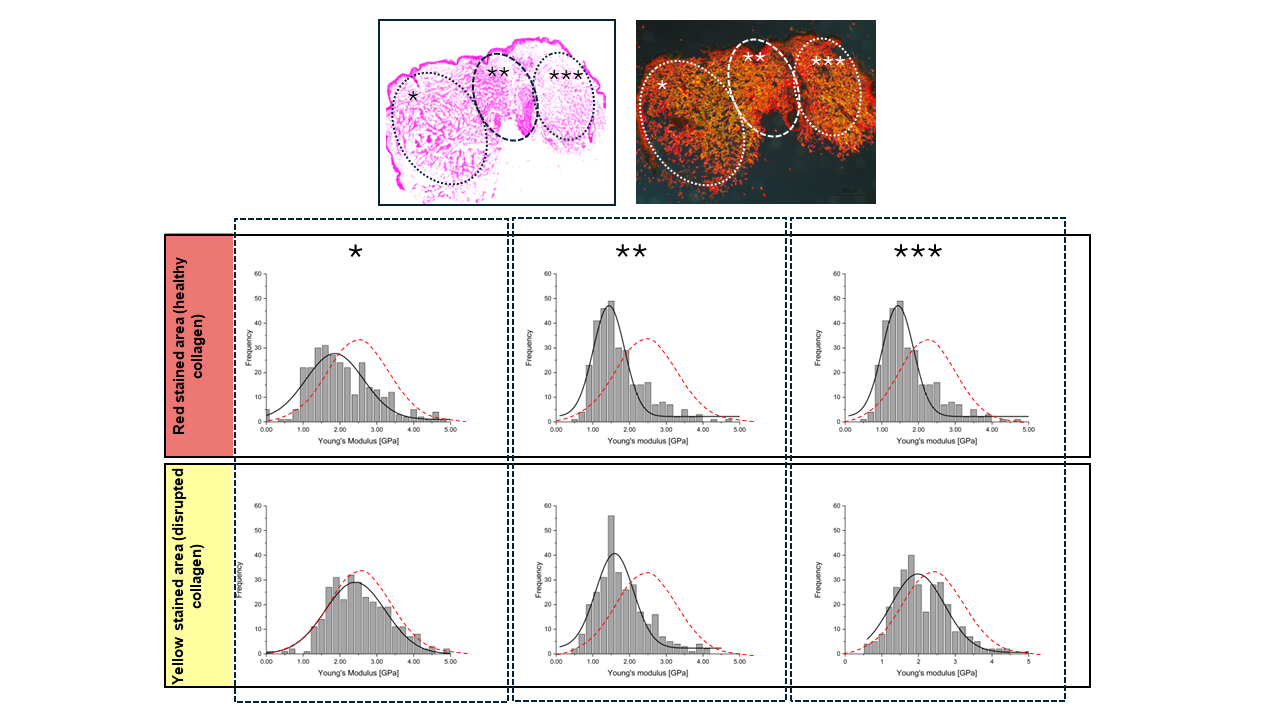

Supplement: S1 Fig — A unimodal distribution of Young’s moduli was observed for both normal and disrupted collagen across all three regions. The red dotted line represents the distribution of healthy collagen in the control group. (TIF) [file pone.0307442.s002.tif]
