# Supplementary material for: Investigation of dermal collagen nanostructures in Ehlers-Danlos Syndrome (EDS) patients
Source: PLoS One. 2024 Aug 22;19(8):e0307442. doi: 10.1371/journal.pone.0307442 (PMC11341037; doi:10.1371/journal.pone.0307442)
Supplement: Attachment — Submitted filename: response letter.docx [file pone.0307442.s003.docx]

**To the Editor and the Reviewer,**

The research team would like to acknowledge and thank the two reviewers for reviewing our manuscript and providing us with constructive feedback, which has been addressed below:

1. **The authors describe the use of genetic testing, including EDS related gene panel, in the studied individuals.
   Please provide the list of genes included in the aforementioned panel and include the molecular details and ACMG classification of the COL1A1 variant identified in the cEDS case.**

Thank you for your comment. The standard EDS related comprehensive gene panel was done at our institute employs Next generation Sequencing (NGS) with duplication and deletion analysis by exon targeted microarray. This test includes 22 genes. Details can be found online on the website (https://www.sickkids.ca/siteassets/care--services/for-health-care-providers/lab-information-sheets/connective-tissue-disorders-ehlers-danlos-syndrome.pdf). In the cEDS case, patient was found to have a pathogenic variant (c.1053delG in exon7) of the COL5A1 gene.

The body of the manuscript has been edited accordingly (as follow).

Section:

Clinical cases: Next generation Sequencing (NGS) with duplication and deletion analysis by exon targeted microarray was performed at SickKids hospital for diagnosis. This test includes 22 genes. Details can be found online on the SickKids website.

Study ID No 5 (labeled as cEDS throughout the paper)

Genetic testing revealed a COL1A1 mutation (c.1053delG in exon7), confirming the diagnosis of classical EDS.

**2.** **The authors show skin biopsy of the hEDS-scleroderma patient to display patchy abnormalities in terms of collagen fiber density with three different regions that could be identified by light microscopy, while the one with the highest fiber density was ascribed to scleroderma related changes. It seems prudent to analyze the AFM findings including collagen fiber elasticity measurements separately for each one of these three regions of the hEDS-scleroderma patient.
This seems important since previous paper utilizing AFM including Young’s Modulus measurements in the setup of scleroderma showed increased collagen fiber stiffness (PMID 28138238). This point should be discussed.**

AFM images (5x5 µm) were acquired from three normal and three disrupted areas (guided by PS image) of the histological section of the skin biopsy. Subsequently, a total of 300 force curves were recorded for each of the normal and disrupted areas. A unimodal Yong’s moduli distribution was observed for both normal and disrupted collagen in all the three regions similar to hEDS-Scleroderma curves presented in Figure 3A. Suggesting that in each of these regions, a single population of collagen fibrils is present. These mechanical data must be interpreted carefully, as we only had one case with both EDS and scleroderma. Further association of the mechanical findings and H&E staining requires more skin tissue from similar patients

These findings have been added as supporting information to the manuscript. And the body of the manuscript on the section “Collagen fibrils elasticity to determine EDS phenotype? “, has been edited as follow: “Additionally we have plotted the Yoong’s moduli distribution for all the three regions denoted as (*, **, **) and found similar distribution in all the three regions as shown in S1Fig”

**Figure S1** Shows the distributions of Young's moduli of collagen fibrils recorded in the *, **, and ** regions of the hEDS-Scleroderma case. A unimodal distribution of Young's moduli was observed for both normal and disrupted collagen across all three regions. The red dotted line represents the distribution of healthy collagen in the control group

**3. The details of full clinical and laboratory findings of the hEDS-scleroderma patient should be included, with the emphasis on the clinical scleroderma subtype, autoantibodies present and disease duration.**

We thank you for the suggestion. The focus of this paper is to highlight the molecular histophysiologal profile of collagen fibrils and to keep the manuscript succinct, we had not included significant details of the clinical phenotype. We have made modifications and included the relevant clinical and laboratory findings, duration and autoantibodies. We have edited the body of the manuscript under the section, clinical cases - Study ID No 4 (labeled as hEDS- Scleroderma throughout the paper) “patient had a 10-year history of inflammatory arthralgias involving PIP and MCP joints, diffuse muscle and joint pains affecting multiple areas of body, secondary diagnosis of fibromyalgia, patchy areas of skin thickening, and Raynaud’s phenomenon. History was negative for uveitis, iritis and psoriasis. Lab testing was positive for Anti-Nuclear Antibody (ANA) and Anti centromere Antibody (ACA) and negative for Anti CCP and rheumatoid factor.”

4. **The existing literature addressing the previous use of AFM for connective tissue disorders should be included in introduction.**

We thank the reviewer for the comment. We have included the following in introduction:

AFM has previously used for connective tissue morphometry studies such as Study of the Extracellular Connective Tissue Matrix in Patients with Pelvic Organ Prolapse [34], nanohistological investigation of scleroderma [35], oral submucous fibrosis [36], assessments of extracellular matrix in intervertebral disc and degeneration [37], and many other studies. In this study we have employed AFM to examine ex-vivo skin biopsies obtained from EDS patients to characterize collagen morphometric differences in cEDS and hEDS.

5. minor comments:
**page 5 line 79 - "genetic variants of EDS" while implying additional genes causative of EDS. Please rephrase**

Thank you for the comment. The sentence has been edited as follow “hEDS shares several clinical features with EDS subtypes but lacks confirmed genetic markers”

**page 5 line 85 – typo, “relayed” should be “related”**

We are sorry for the type. It is now corrected.
